# Infrared Ion Spectroscopy of Gaseous [Cu(2,2′-Bipyridine)_3_]^2+^: Investigation of Jahn–Teller Elongation Versus Compression

**DOI:** 10.1021/acs.jpca.4c07019

**Published:** 2025-01-28

**Authors:** Musleh Uddin Munshi, Giel Berden, Jos Oomens

**Affiliations:** † Department of Chemistry, Sogang University, Seoul 04107, Republic of Korea; ‡ Institute for Molecules and Materials, FELIX Laboratory, Radboud University, Toernooiveld 7, 6525 ED Nijmegen, The Netherlands; § University of Amsterdam, Science Park 904, 1098XH Amsterdam, The Netherlands

## Abstract

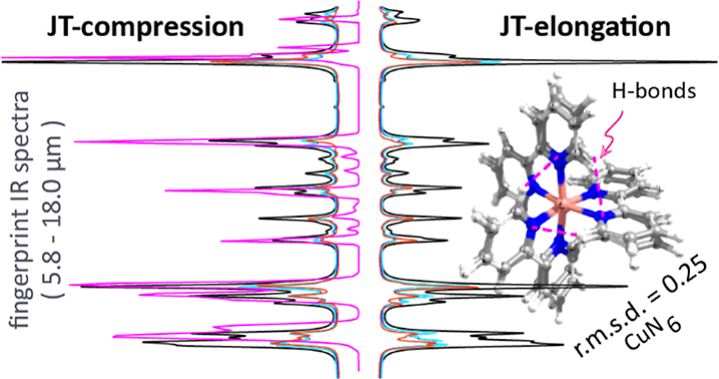

Symmetry breaking is ubiquitous in chemical transformations and affects various physicochemical properties of materials and molecules; Jahn–Teller (JT) distortion of hexa-coordinated transition-metal–ligand complexes falls within this paradigm. An uneven occupancy of degenerate 3d-orbitals forces the complex to adopt an axially elongated or compressed geometry, lowering the symmetry of the system and lifting the degeneracy. Coordination complexes of Cu^2+^ are known to exhibit axial elongation, while compression is far less common, although this may be due to the lack of rigorous experimental verification. Here, we present the gas-phase vibrational spectrum of the archetypal [Cu(2,2′-bipyridine)_3_]^2+^ ionic complex, obtained by infrared multiple-photon dissociation (IRMPD) spectroscopy using the widely tunable IR free-electron laser FELIX. Predicted vibrational spectra at the density functional level of theory are nearly—but not entirely—identical for the two JT-distorted geometries. We compare experimental and theoretical spectra and address the question of an axially elongated or compressed geometry of the complex, or a mixture thereof, in the gaseous ion population.

## Introduction

The tris-2,2′-bipyridine copper(II) complex, [Cu(bpy)_3_]^2+^, has been an archetypal metal–organic system in coordination chemistry, just as its ruthenium analog, [Ru(bpy)_3_]^2+^. Unlike other transition metals, the hexacoordinated Cu(II) complex is known to exhibit extreme Jahn–Teller effects (JTE).^1−5^ In a nonlinear molecule, if degenerate molecular orbitals (MO) are asymmetrically occupied, a distortion occurs, lowering the symmetry and thus removing the degeneracy. Although the theorem was limited originally to molecules, it was extended to ions in crystals by Van Vleck in 1939.^6,7^ Physical and chemical properties of molecules or materials are affected by the JTE, especially the electron distributions, as well as the spatial arrangement of the involved atoms. For this reason, conductivity, magnetism, and many other physical properties of (ceramic) materials are substantially affected. Generally, similar distortions occur in molecular systems, such as organic radicals, fulleride anions, and ionic carbon nanotubes.^8^ Similarly, closely related effects (Peierls distortions) have been observed in linear one-dimensional systems, solid lattices, polymers, and chains of atoms on surfaces.^9,10^

**Figure 1:**
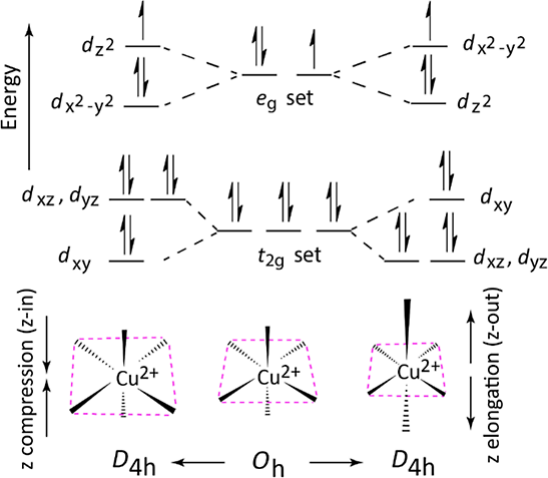
Jahn–Teller distortions of an octahedral Cu(II) complex where geometrical distortions lower the energies of the complexes with uneven occupancies of the e_g_ orbitals. (Adapted from ref (3) with permission from the Royal Society of Chemistry, Copyright 2013, link.)

For the first-row transition metal complexes, the JTE is induced by the uneven occupancy of the metal 3d-subshell in a ligand field that partly removes the degeneracy that exists in the free metal ion. For instance, depending on the spin multiplicity, orbitally degenerate electron configurations are possible for partially filled d-orbitals i.e., d^1^–d^9^ in octahedral symmetry (O_h_) with exceptions for d^3^, d^5^ (high spin), d^6^ (low spin), d^8^. The energy levels rise as the six ligands approach the metal ion to form an octahedron as shown in Figure 1. Moreover, energy levels split into two groups having symmetry e_g_ (, d_*z*^2^_) and t_2g_ (d_*xy*_, d_*yz*_, d_*xz*_), since the orientations of the e_g_ orbitals are such that they experience a comparatively higher repulsion of the head-on approaching ligands than the t_2g_ orbitals, which lie in between ligands. Thus, uneven occupancy of the e_g_ set is often affected the most and uneven occupancy in the t_2g_ set shows weak JT distortion. Large JT effects are observed in six-coordinate complexes of high-spin d^4^ Cr(II), Mn(III), low-spin d^7^ Co(II), and d^9^ Cu(II) ions. Among investigated JTE systems, six-coordinate Cu(II) complexes are by far the most common. Although elongation as well as compression geometries are possible, the preferred direction has mostly been identified as elongation,^3,11−15^ while tetragonal compression has not been reported, perhaps due to the lack of proper experimental verification.^3^ In general, many observed structural, and spectroscopic properties remain incompletely understood, while numerous theoretical predictions remain yet to be verified due to the lack of adequate experimental data^16,17^ on model systems.

Recently, Weber and co-workers^12^ studied [M^2+^(bpy)_3_]^2+^ (M = Mn, Fe, Co, Ni, Cu, Zn) ions at cryogenic temperature (25 K) in vacuo using an ion storage mass spectrometer coupled with photodissociation spectroscopy, where mass-isolated ions are irradiated with wavelength-tunable UV/vis laser radiation. Comparison of the gaseous UV spectra with those obtained in the condensed phase brought interesting insights. Although band shapes were compatible with those in aqueous solutions and in crystals at room temperature, bands in the condensed-phase spectra were red-shifted and broadened. The shift was attributed to the stabilization of the excited states in solution. Despite the elimination of solvents, broadness remained because of the short lifetime of the excited state and overlapping Franck–Condon envelopes and high electronic density of states. Theoretical calculations based on time-dependent density functional theory (TDDFT) located distinguishable π–π* transitions, and some transitions involve orbitals with significant density on Cu.^12^ Nonetheless their observations indicate that additional factors perhaps are responsible for the limited spectral resolution and computations agree only qualitatively.

Besides UV/vis spectroscopy,^12,18−21^ nuclear magnetic resonance (NMR),^22^ electron spin resonance (ESR),^13,23^ and infrared (IR) spectroscopy^22,24−26^ have been extensively utilized to study hexa-coordinated transition metal complexes to investigate the JTE. Theoretical modeling^2,16,27−29^ was employed to rationalize the observed spectral features, especially the intrinsic d*–*d transitions. Theoretical advances rely strongly on the availability of experimental data on model systems with known absolute configurations, preferably in complete isolation. Gas-phase data is scarce for coordination complexes of transition metal ions in both tetrahedral and octahedral ligand environments. It is worth noting that it remains notoriously challenging to predict structural and spectroscopic properties of transition metal–ligand complexes due to the near degeneracy of the unevenly occupied d-orbitals, such as in hexa-coordinated Cu^2+^ (d^9^).

Ion storage mass spectrometry (MS) offers an excellent means for investigating charged gaseous complexes in isolation, removing external influences. Recently we investigated hexa-coordinated complexes of transition metal ions with similar organic ligands for structural characterization using infrared multiple-photon dissociation (IRMPD) spectroscopy.^30−33^ This combines MS with the structural sensitivity of vibrational spectra. Three-dimensional structures can be derived from theoretical interpretations of the IR spectra, revealing the spin multiplicity of the system and the potential presence of additional higher-energy conformers.^30,31^

**Figure 2:**
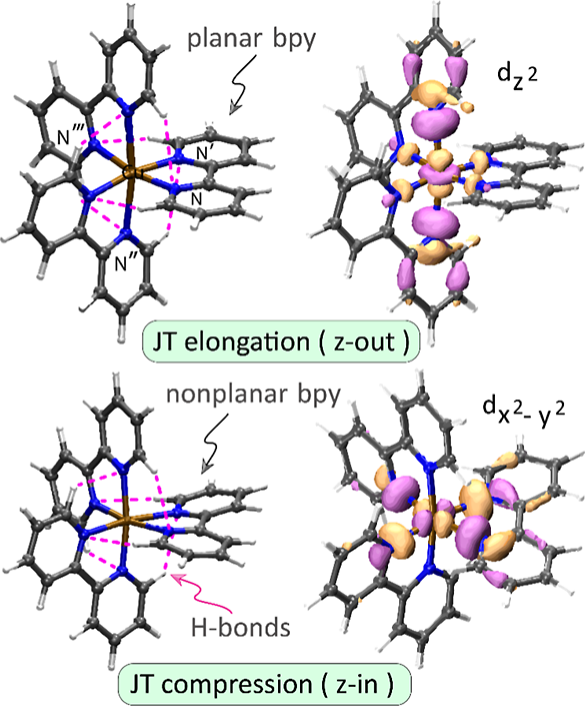
Optimized structures of the Λ-[Cu(bpy)_3_]^2+^ isomer: (top) minimum-energy electronic ground state and (bottom) higher-energy conformer, with the singly occupied molecular orbital (SOMO) showing d_*z*^2^_ and  character, respectively. Coordinate labeling is shown in the top panel. H-bonds are represented with dashed lines and swapping of one planar ligand to one nonplanar ligand is indicated.

Here, we record the IR spectrum of the gaseous mass-to-charge (*m*/*z*) isolated [Cu(bpy)_3_]^2+^ complex (Figure 2) to investigate its geometrical and electronic structure. The complexes are isolated in a Paul-type quadrupole ion trap (QIT) MS^32,34^ coupled to the wavelength tunable infrared free electron laser FELIX,^35−37^ where we probe the vibrational spectrum of the complex by monitoring the IR frequency-dependent photofragmentation across the fingerprint range (600–1700 cm^–1^). Detailed theoretical analyses of the experimental spectrum assess the potential coexistence of JT elongation and compression conformers.

## Methods

### IRMPD Action Spectroscopy

Experiments were performed in a modified 3D-quadrupole ion trap mass spectrometer (QIT MS, Bruker, AmaZon Speed ETD, Bremen, Germany), which has been described in detail elsewhere.^32,34^ The cation of interest, [Cu(bpy)_3_]^2+^, was generated by electrospray ionization (ESI) starting from a solution containing equimolar (1 μM) amounts of Cu(NO_3_)_2_ salt and 2,2′-Bipyridine in 1:1 MeOH/H_2_O. IRMPD spectra were recorded from 600 to 1700 cm^–1^ using wavelength tunable infrared radiation from the FELIX free electron laser (FEL).^38,39^ FELIX was operating at a repetition rate of 10 Hz while producing 6 μs-long macropulses with energies up to 90 mJ per pulse.

ESI generated ions were accumulated for 30 ms before irradiation with FELIX. The isotopically pure dication [Cu(bpy)_3_]^2+^ at *m*/*z* 265.5 was mass-isolated, retaining only the 63-isotope of copper. These ions were irradiated with two FEL macropulses having a maximum pulse energy of 45 mJ. Every 5 cm^–1^ in the wavelength scans, six mass spectra were averaged. One major fragment at *m*/*z* 187.5 (loss of one bpy unit) was observed upon irradiation, along with several minor fragments at *m*/*z* 236, 219, 157, and 156. Note that we reported similar products upon collision induced dissociation (CID), electron transfer dissociation (ETD), and photofragmentation for [Cu(bpy)_2_]^2+^ (*m*/*z* 187.5).^40^

Mass selected molecular ions were vibrationally excited whenever the laser frequency was in resonance with one of the normal-mode frequencies of the ion. Multiple photons are absorbed and statistical redistribution of the absorbed energy promotes the increase of internal energy of the ions via intramolecular vibrational redistribution (IVR).^41,42^ Once the energy exceeds the lowest energy dissociation threshold, the molecular ion undergoes unimolecular dissociation. IR spectra were generated by plotting the natural logarithm of the fragmentation yield of the precursor ions as a function of laser frequency.^31,33,43,44^
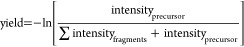


The fragmentation yield was linearly corrected for frequency-dependent variations in the laser pulse energy and a grating spectrometer was used to calibrate the IR frequencies.

### Computational Modeling

Geometries were optimized as isolated molecules at the density functional level of theory (DFT) using conventional hybrid B3LYP^45,46^ and long-range corrected CAM-B3LYP^47^ functionals with the Def2TZVP^48^ basis set. Even though the provided initial geometry was the same, having D_3_ symmetry, B3LYP optimization resulted in axial elongation while CAM-B3LYP led to axial compression (Figure 2). It is noteworthy that when these optimized geometries are swapped and used as initial guess for the other level of theory, the resulting geometries remain the same. B3LYP geometries were also optimized in water using the polarizable continuum model (PCM). Computed (linear) harmonic IR frequencies were scaled by factors of 0.984 (B3LYP) and 0.964^49,50^ (CAM-B3LYP) to compensate for anharmonicity and basis set incompleteness, such that an observed IRMPD band at 1015 cm^–1^ was reproduced. Results of CAM-B3LYP/Def2TZVP are used throughout the discussion, unless indicated otherwise. The Gaussian16^51^ computational program package was employed for these computations. In addition, the Amsterdam density functional (ADF) program package^52,53^ was used, where a GGA (generalized gradient approximation) functional (BP86) was chosen with uncontracted Slater type orbitals (STOs) of triple-ζ quality, including two sets of polarization functions (TZ2P^52^).

No imaginary frequencies were found, confirming that the stationary points were true minima on the molecular potential energy hypersurface. Scaled frequencies were convoluted using a 10 cm^–1^ full-width at half-maximum Lorentzian line shape function to facilitate experimental versus theoretical spectral comparisons.

The Multiwfn (v 3.8) program package^54^ was used to compute the partial vibrational spectra (PVS), which requires molecular wave function data as input. Similar to vibrational energy distribution analysis (VEDA),^55^ the vibAnalysis^56^ computer program was used as potential energy decomposition (PED) scheme for elucidation of the spectra, including the calculation of vibrational mode automatic relevance determination (VMARD). Furthermore, vibrational frequencies of conformers were visualized with the aid of the PyVib2 1.0 computer program.^57^

## Results

### Structural Parameters of [Cu(bpy)_3_]^2+^

Besides the minimum-energy “elongated” conformer, a slightly higher-energy “compressed” conformer is also optimized as shown in Figure 2. Relative energies (Table 1) and key structural parameters (Table 2) are summarized. The structures retain C_2_-symmetry with Cu(II) in a pseudo-octahedral CuN_6_ coordination geometry.

**Table 1:** Calculated Energy Difference between Conformers and the Corresponding Boltzmann Populations at 298 K

	geometry (conformers)	Gibbs free energy/kJ mol^–1^	population (%)
CAM-B3LYP/Def2TZVP (gas)	elongation	0	94
	compression	6.8	6
B3LYP/Def2TZVP (gas)	elongation	0	94
	compression	6.7	6
BP86/TZ2P (gas)	elongation	0	96
	compression	7.9	4
B3LYP/Def2TZVP (water)	elongation	0	91
	compression	5.8	9

**Table 2:** Average Cu–N Distances (Å) and N–Cu–N Bond Angles (°) for [Cu(bpy)_3_]^2+^ Compared with Available X-ray Crystallographic Data.^58−61^^a^

	B3LYP/Def2TZVP		CAM-B3LYP/Def2TZVP		X-ray crystallography	
parameter	elongation	compression	elongation	compression	ref (60)	ref (59)
Cu–N (axial)	2.401(0)	2.042(0)	2.365(0)	2.025(0)	2.345(0.150)	2.186(0.030)
Cu–N (equa.)	2.088(0.011)	2.256(0.047)	2.067(0.009)	2.229(0.042)	2.028(0.009)	2.085(0.014)
Bond angle (deg)						
∠N–Cu–N′	75.8(2.9)	75.9(3.0)	76.3(2.8)	76.4(2.9)	77.3(3.2)	
∠N–Cu–N″	91.6(0.9)	91.6(2.5)	91.4(0.8)	91.7(2.7)	88.8(4.7)	
∠N–Cu–N‴	169.1(0.4)	169.0(1.8)	169.5(0.8)	169.3(1.6)	171.6(4.7)	170.2
∠N′–Cu–N″	96.7(1.3)	96.6(0.4)	96.5(1.5)	96.4(0.5)	97.1(3.9)	

a Used atom labels are shown in Figure 2. Parenthesized values are calculated standard deviations.

The elongated conformer shows a pair of long axial Cu–N bonds (2.365 Å) and four shorter equatorial bond distances (2.067 Å). The significant axial elongation as compared to the equatorial Cu–N bond distances confirms the JTE of this classical complex with d^9^ electronic configuration in an octahedral ligand environment. Additional structural anomalies are observed in the average bond angles. For instance, the ligand bite angles (∠N–Cu–N = 76.3° ± 3°) are not as regular as in a complex without JTE (e.g., [Ru(bpy)_3_]^2+^).^33^ Furthermore, two axial or equatorial N atoms remain nonlinear, which is reflected in the through-complex N–Cu–N bond angles of 169.5° ± 0.8°, deviating significantly from 180°. Equatorial N atoms are nonplanar (∠NNNN = 13.4°).

In Table 2, we notice variations in the X-ray structural parameters reported for the solid salt, which may be related to the presence of different counteranions (TaF_6_^–^, BF_4_^–^, PF_6_^–^) and temperatures (100, 203, and 291 K).^58−60^ In contrast, our gas-phase experiment is conducted for the isolated Cu(II) coordination complex without any counteranion. Therefore, the related data can be considered as pure, and the observed phenomena would be intrinsic to the electronic configuration alone.

In comparison to X-ray crystallographic data,^58−60^ the calculated average Cu–N axial distance of 2.365 Å is only 0.02 Å larger than that of the experiment (average 2.345 ± 0.150).^60^ Similarly, the equatorial Cu–N bonds (2.067 Å) are close to experiment (2.028 ± 0.009).^60^ Similar axial elongation (2.406 Å) was observed also for the hexa-aqua Cu(II) complex in the solid phase.^62^

Calculated ligand bite angles show excellent agreement with X-ray^60^ data (Table 2). The average calculated ligand bite angle (76.3° ± 2.8°) agrees with experimental values^60^ (77.3° ± 3.2°). In addition, the largest ligand bite angle (∠N–Cu–N = 79.6°) occurs for the bpy ligand that has dihedral angle (between two rings) that is smaller (∠N–C–C–N = 0.5°) than the other two bpy ligands (∠N–C–C–N = 13.7°). This enhances the π-electron resonance and may be reflected in the relevant normal-mode frequencies (e.g., bpy ring breathing). These structural differences are indeed supported by the experimental IR spectra and their match with calculations (vide infra).

The optimized geometry of the axially compressed conformer exhibits two short Cu–N (2.025 Å) bonds along the *z* axis and two medium (2.192 Å) as well as two long (2.266 Å) bonds in the *xy* plane. Note that these long bonds are associated with a single bpy ligand, which is significantly less planar—reflected in the dihedral angle (∠N–C–C–N = 14.0°)—than the other two bpy ligands (∠N–C–C–N = 3.7°). Careful inspections show that both N atoms of this nonplanar bpy are in closer proximity to the H atoms of the neighboring bpy ligands due to axial compression. On top of this, the nonplanarity of the bpy ligand enhances two H-bonds (N···H = 2.5 Å) as compared to the other H-bonds (N···H = 2.7 Å). These rather strong H-bonds distort this bpy ligand further from planarity. These results clarify cooperative bond dynamics between conformers: for axial elongation, equatorial N-atoms remain closer to the Cu center and vice versa for axial compression. Thus, strictly, conformer elongation approaches a tetragonal elongated system, while conformer compression resembles a pseudo-orthorhombic system, where all three axes are unequal in length and not orthogonal.

### Ligand Shape Analysis

An interesting observation is that the Cu–N distances influence dihedral angles in the respective bpy ligand, where the ligands become more planar as their Cu–N distance becomes shorter. Note that the ∠N–C–C–N dihedral angle of the free neutral ligand varies between 39° and 180° for *cis* and *trans* conformers (Supporting Information Figure S1). We find two nonplanar (∠N–C–C–N = 13.9°) ligands with JT elongation, whereas a single nonplanar bpy (∠N–C–C–N = 14.6°) is present for the JT compression conformer (Figure 2). The nonplanar ligands remain relatively far from the Cu(II) center as compared with the planar ligands. The ligand distortion thus appears highly sensitive to metal–ligand binding strengths. The ligand planarity is dictated by a competition between maximizing the metal–ligand binding energy, while minimizing the ligand–ligand repulsion, as also explained by Rannulu and Rodgers^63−65^ in collision-induced dissociation MS/MS studies of the complexation of bpy with monocationic Cu, Ni, and Zn.

## Analyses of the IR Spectra of [Cu(bpy)_3_]^2+^

### Overview

**Figure 3:**
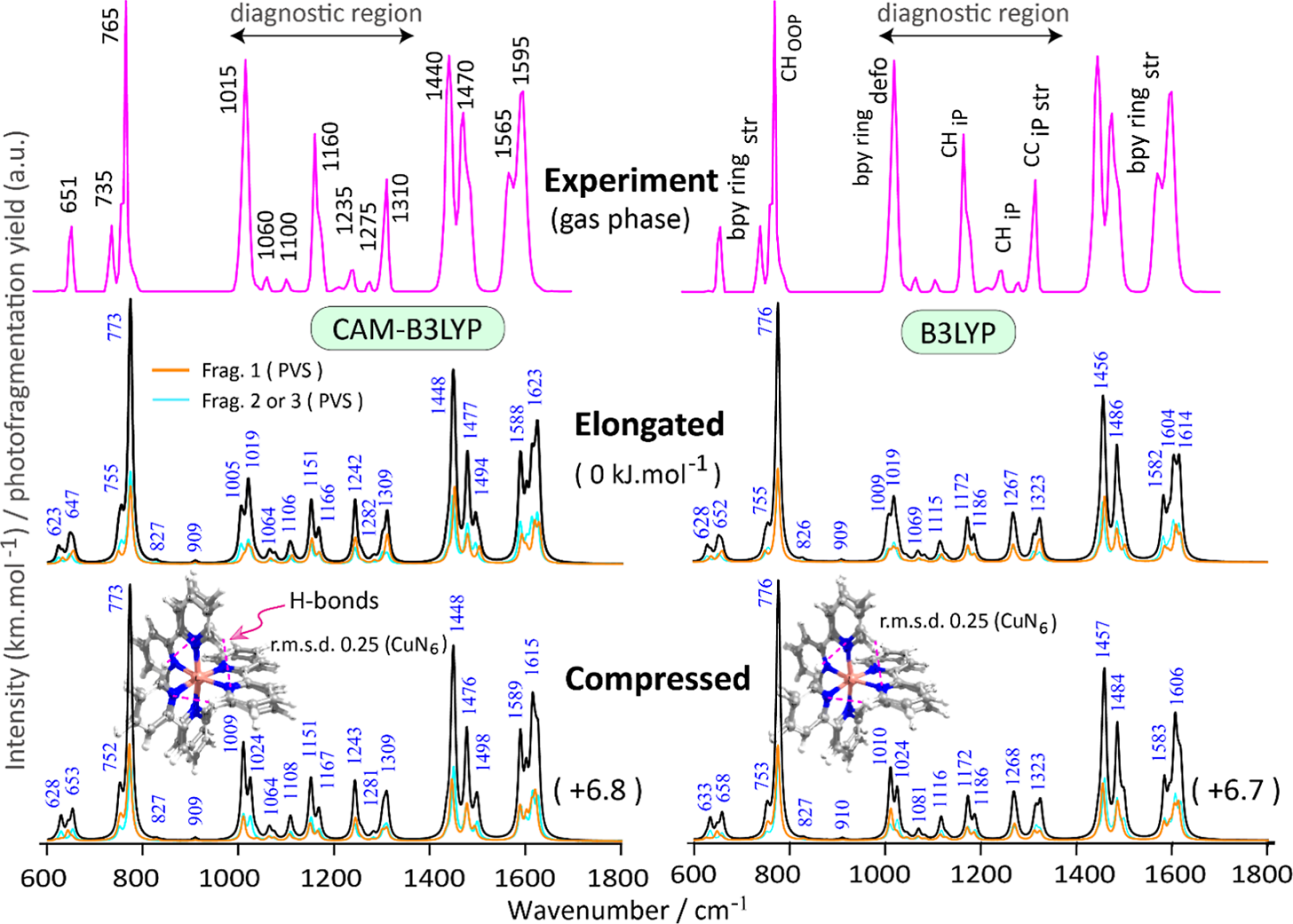
IRMPD spectrum with approximate vibrational mode frequencies and characters of [Cu(bpy)_3_]^2+^ compared with the calculated IR spectra at CAM-B3LYP/def2TZVP (left) and B3LYP/def2TZVP (right) levels. Partial vibrational spectra (PVS) are computed for the coordinated bpy ligands (fragment 1, 2, or 3) to distinguish planar and nonplanar ligands. Optimized structures of elongated and compressed conformers are merged and displayed in the lower panels together with the r.m.s.d. value for the CuN_6_ sphere only. Despite the geometrical/spectral similarities, the PVS for planar and nonplanar ligands, represented by the colored (orange, cyan) traces, provide key differences between conformers, especially around 1000 cm^–1^.

Figure 3 shows the experimental IRMPD spectrum of [Cu(bpy)_3_]^2+^ containing several well-resolved vibrational bands. Calculated linear IR spectra of the two JT-distorted conformers are shown for comparison with experiment. In general, the conventional B3LYP functional has been highly successful in accurately predicting observed IR band positions.^66^ This is also the case here, although a few bands remain unexplained, especially in the 1200–1350 cm^–1^ range. CAM-B3LYP results show some improvement in reproducing the band positions in this range, although predicted bands above 1400 cm^–1^ are shifted slightly too far to the blue. A few predicted low-intensity (<2 km mol^–1^) IR bands (800–950 cm^–1^) remain unobserved because IR excitation does not reach the IRMPD threshold at these frequencies.^33,40,67^ This complex appears to be relatively fragile, requiring only low pulse energies for photofragmentation to occur, which gives confidence in our IRMPD spectrum and the ability to observe even weak features, unlike some other systems.^68^ Overall, this yields an experimental spectrum with well-resolved and narrow-bandwidth features of nearly “single-photon” quality.^69,70^

In addition to the computed IR spectra, partial vibrational spectra (PVS)^54^ are calculated for each of the coordinated bpy ligands for both conformers. The PVS reveal the individual ligand contributions to the overall vibrational spectrum (Figure 3). Ligands can be categorized as planar or nonplanar based on the dihedral angles. The elongated conformer has one planar bpy and two nonplanar ones, whereas the compressed conformer has two nearly planar and one nonplanar bpy ligand. Due to prominent variations of the dihedral angles of bpy, their PVS show slight differences and form sensitive markers for each of the two conformers. Computed spectra of the two conformers in the 1400–1700 and 600–800 cm^–1^ ranges are nearly identical and therefore less useful for conformer profiling. These regions present strong absorption bands that are red-shifted compared to gaseous IR spectra of neutral bpy, reported in the NIST Chemistry Webbook (see Supporting Information Figure S1). In contrast, bands in the 900–1400 cm^–1^ region show more diagnostic value. Especially around 1000 cm^–1^, the PVS for the planar bpy in the elongated conformer (orange trace) shows a band at an IR frequency that is about 10 cm^–1^ higher than in the compressed conformer, where the same ligand becomes nonplanar. The shape of the observed feature near 1015 cm^–1^ therefore reflects the ratio of planar and nonplanar bpy ligands present and is of interest to assess the presence of elongated and compressed conformers in the ion population. We inspect this part of the spectrum in more detail below.

**Figure 4:**
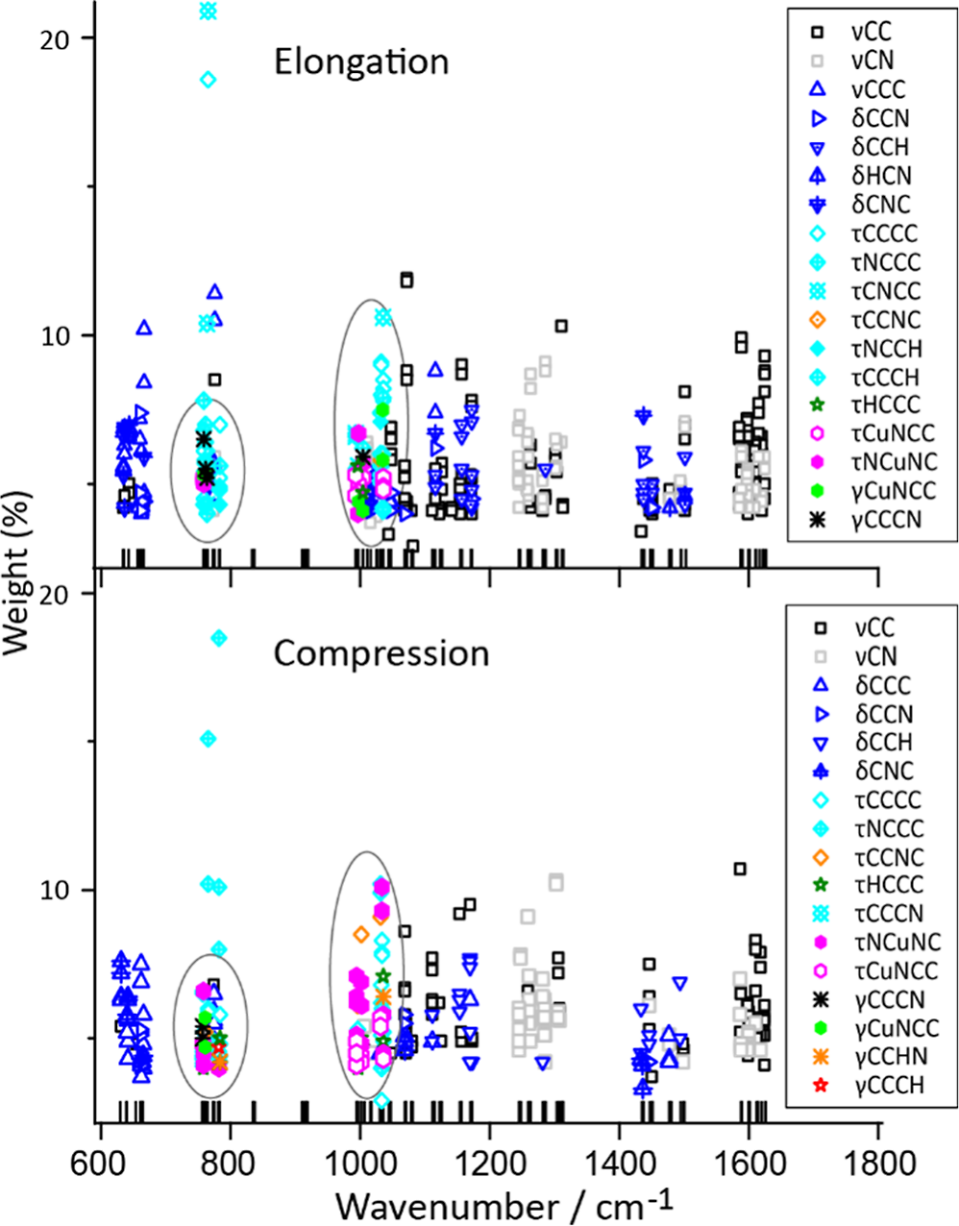
Distribution of the internal coordinates as relative weights contributing to the vibrational normal modes, calculated using vibrational mode automatic relevance determination (VMARD):^56^ stretching (υ), bending (δ), torsion (τ), and out-of-plane (γ) deformations are defined based on the involved number of atoms. Modes strongly involving the metal ion are indicated in the ellipses.

For further elucidation of the IR spectra, we employed the vibAnalysis computer code, which implements vibrational mode decomposition (VMD),^56^ similar to potential energy decomposition (PED),^55^ where calculated normal modes are presented as a superposition of local modes involving two, three, or four connected atoms, giving rise to stretching, bending, and torsion/out-of-plane vibrations, respectively (Figure 4). Despite numerous overlaps, mode types including related atoms become evident and assist in localizing, for instance, vibrations involving the metal ion around 1000 and 770 cm^–1^.

### Jahn–Teller Elongated Conformer

First, we quickly analyze the experimental spectrum in Figure 3 in the less diagnostic 1400–1700 and 600–900 cm^–1^ ranges in terms of the theoretical IR spectrum for the lowest-energy conformer (elongation). Bands in the high-frequency range mostly involve ring vibrations of the bpy ligands. IR band maxima are observed at 1595 and 1565 cm^–1^, matching with the predicted bands at 1623 and 1588 cm^–1^. Similarly, the observed band maxima at 1470 and 1440 cm^–1^ are calculated well at 1477 and 1448 cm^–1^. Previously, we reported IR spectra for the redox pair [Cu(bpy)_2_]^2+/+^ using the same QIT MS apparatus;^40^ band positions and mode characteristics were found to be similar to those reported here. In the low frequency region, three band maxima are observed at 765, 751, and 635 cm^–1^, as predicted reasonably well by bands at 773, 755, and 647 cm^–1^ corresponding to butterfly, CH out-of-plane and CH in-plane vibrations, respectively. In addition, a band (shoulder) is barely observed at 627 cm^–1^, which is calculated at 623 cm^–1^. Overall, relative intensities of the observed bands are in reasonable agreement with the prediction.

We now turn our attention to the 900–1400 cm^–1^ range, where band assignments involve mostly CH out-of-plane bending, CH in-plane bending and bpy ring breathing. Three relatively high-intensity bands and some weak bands are observed. The predicted band at 1309 cm^–1^ is measured at 1310 cm^–1^ and the relatively broad band observed at 1160 cm^–1^ is explained by two predicted bands in close proximity at 1151 and 1166 cm^–1^. Two low-intensity bands are observed at 1100 and 1060 cm^–1^, which are predicted at 1106 and 1064 cm^–1^.

The most interesting band in the context of this study is observed at 1015 cm^–1^ (fwhm 16 cm^–1^) that involves stretching of the Cu–N bonds in the molecule. A similar experimental band was previously observed at 1020 cm^–1^ in the tetra-coordinated distorted-square planar [Cu(bpy)_2_]^2+^ complex.^40^ In the hexa-coordinated [Cu(bpy)_3_]^2+^ studied here, this band shifts to 1015 cm^–1^ due to the weaker Cu–N bonds upon attachment of an extra bpy ligand.^63,64^

Figure 3 shows that the computed spectra predict a partially resolved double band structure with asymmetric relative intensities of the two components that reverse from the elongated to the compressed conformer. The underlying PVS indicate that the difference in the asymmetry of the band shape between the two conformers originates in the different contributions from the three bpy ligands, where the elongated conformer has one planar and two nonplanar bpy ligands, and vice versa for the compressed conformer. In Figure 3, orange traces represent PVS for planar ligands and cyan traces represent PVS for nonplanar ligands.

**Figure 5:**
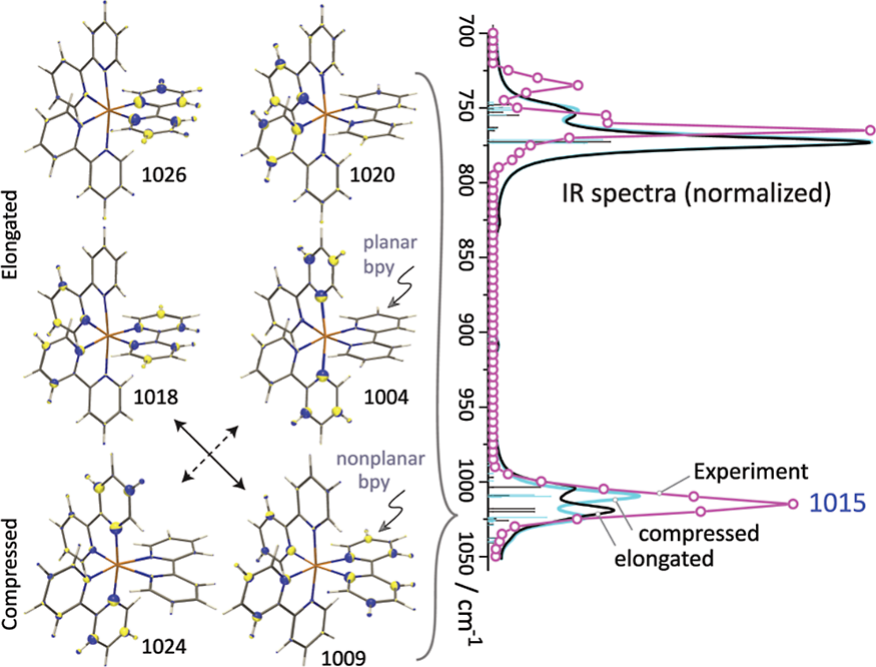
(Left) Vibrational displacements^57^ for the normal modes contributing to the diagnostic IR band observed at 1015 cm^–1^, giving rise to Cu–N bond stretching motion. The blue and yellow colors represent the direction of the displacement vectors, while the volume of the spheres correlates with the amplitude of the atomic nucleus. Double headed arrows connecting the elongated and compressed structures indicate a mode character “swap”. (Right) Zoom in of the IR spectrum. Computed frequencies are scaled by 0.964 to match the observed band at 1015 cm^–1^.

For the elongated conformer, the double-band structure has predicted maxima at 1005 and 1019 cm^–1^, resulting from the convolution of several underlying normal modes at 1004, 1009, 1018, 1020, and 1026 cm^–1^ (see Figure 5). The relative spacing of these modes qualitatively agrees with the width of the observed band, but not the observed band shape. The normal modes correspond to bpy ring breathing vibrations either localized on one ligand or delocalized over multiple ligands that also cause stretching along the Cu–N and N–Cu–N coordinates, as depicted in Figure 5.

The mode computed at 1026 cm^–1^ corresponds to a localized symmetric bpy ring breathing vibration, causing the symmetric stretching of two equatorial Cu–N bonds from the bpy ligand that is significantly more planar (∠N–C–C–N < 1°) than the other two ligands (∠N–C–C–N = 14°), where these dihedral angles agree qualitatively with the X-ray crystallography data.^59^ Contrary to the 1026 cm^–1^ mode, the mode predicted at 1018 cm^–1^ involves N–Cu–N stretching of all equatorial Cu–N bonds, while the other two rings attached axially remain nearly stationary. Just on the opposite side of the planar bpy, the normal mode at 1020 cm^–1^ is delocalized over the two nonplanar bpy ligands and involves symmetric stretching of two of the equatorial Cu–N bonds. The planar bpy facilitates stronger π-electron resonance, so that its mode appears at slightly higher frequency (1026 cm^–1^) than for the nonplanar bpy ligands (1018, 1020 cm^–1^). Also, the Cu–N equatorial bonds to the planar bpy are slightly shorter (0.02 Å) than the other two equatorial bonds. The low-frequency normal mode at 1004 cm^–1^ involves asymmetric axial N–Cu–N stretching. Therefore, it can be considered as a diagnostic band for the axial elongation.^40,63,64^

### Jahn–Teller Compressed Conformer

We now analyze the 1015 cm^–1^ experimental band in terms of the computed spectrum for the compressed conformer. Again, the calculated PVS allow us to distinguish the contributions of planar versus nonplanar bpy ligands to the total spectrum (Figure 3). Figure 5 shows that, analogous to the predicted bands at 1004 and 1018 cm^–1^ for the elongated conformer, similar normal modes are predicted for the compressed conformer, but with a blue shift at 1009 and 1024 cm^–1^ and with a twist that the mode characteristics are swapped. This small frequency shift may contribute to the broadening of the band in the observed spectrum if both conformers coexist.

The axial compression may intuitively be held responsible for the significant 20 cm^–1^ blue shift of the band at 1024 cm^–1^ (relative to axial elongation), see Figure 5. Vice versa, the band at 1009 cm^–1^ is red-shifted due to the significant increase of the equatorial Cu–N bond distances (2.229 Å). The bands at 1009 and 1024 cm^–1^ are the main contributors to the band in the compressed conformer and their splitting is on the order of the experimental bandwidth.

The compressed conformer is calculated to be 6.8 kJ mol^–1^ higher in energy than the elongated conformer (Table 1), so that a Boltzmann population at 298 K suggests a 94% versus 6% ratio of the two conformers (91% versus 9% in water). Clearly, inaccuracies in the computed energy difference of only one or a few kJ mol^–1^, would quickly change this ratio. Spectroscopically, the band near 1015 cm^–1^ is the only feature that could reveal the actual ratio of conformers in the ion population. The zoomed-in comparison of experimental and theoretical spectra in Figure 5 is tantalizing: one is tempted to conclude that an approximately equal contribution of the two conformers would reproduce the observed spectral band shape best, but we must accept that the computed spectral differences are too small to reliably make this call, despite the good quality of the experimental IRMPD spectrum. Moreover, multiple closely spaced vibrational transitions may slightly skew the spectral envelope in an IRMPD spectrum as a consequence of artifacts of the multiple-photon excitation process.^38^^,^^68,71^ We therefore conclude that the presence of the JT-compressed geometry cannot be evidenced based on the present data but can also not be excluded.

### Isomerization Barrier

**Figure 6:**
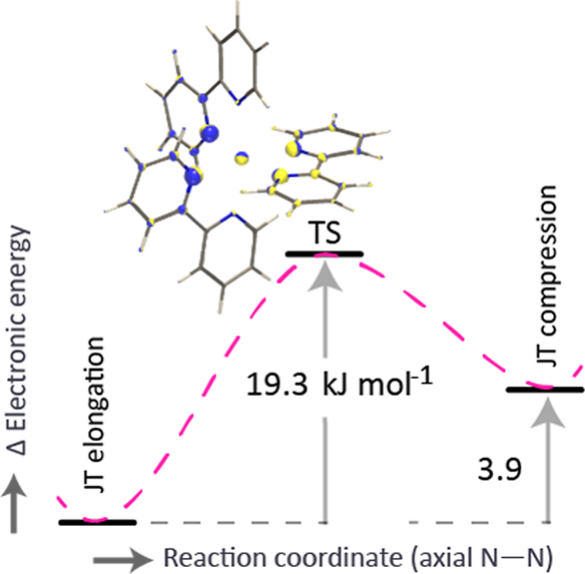
Estimated transition state (TS) barrier based on calculated single-point energies from JT elongation to compression conformers.

Relying on chemical intuition and on single point energy calculations, a transition state (TS) geometry is assumed with near D_3_ symmetry. At the TS, the e_g_ orbitals ( and d_*z*^2^_) are expected to become degenerate. To investigate the influence of orbital occupancy on the TS geometry, MOs with their energies are visualized (Figure S3 in Supporting Information). Swapping of energy levels is indeed observed at the TS between  and d_*z*^2^_ as the molecule switches from compression to elongation. This geometry is further optimized keeping Cu–N distances fixed to 2.170 Å while allowing the rest of the molecule to relax to provide the TS (restricted geometry) as shown in Figure 6. The calculated imaginary frequency is visualized (using the PyVib2^57^ program) and shows the dominant symmetric displacements of the involved nuclei. As expected, the geometry is slightly distorted by structural rearrangement, e.g., ∠N–Cu–N‴ (168.4–170.5°) (Figure 2) and ligand dihedral angles (∠N–C–C–N = 4.0–8.1°). The TS is 19.3 kJ mol^–1^ higher in energy than the JT elongated minimum-energy geometry, so that the thermalization barrier is 15.4 kJ mol^–1^ for the higher-energy compressed conformer (single-point energy of +3.9 kJ mol^–1^). Note that the energy difference between the conformers is known as the warping barrier of the well-known Mexican-hat potential.^16,17^ Due to the TS barrier to thermalization, the higher-energy conformer may remain kinetically trapped^30,44,72^ in the QIT MS. Our theoretical calculations are in line with those of Freitag and Conradie,^2^ who calculated similar electronic energy differences between elongation and compression conformers for a high-spin Mn^III^(β-diketonato)_3_ complex.

## Electronic Structures

### Natural Population Analysis

A natural population analysis (NPA)^73^ was performed giving atomic partial charges via summation over natural atomic orbitals (NAO); key results are summarized in Table 3. JTE can be described based on the natural charges of the CuN_6_ coordination sphere alone. Although the charge on Cu remains very similar for both conformers, larger changes are observed for the nitrogen partial charges, where the axial N-atoms carry the highest charge for the elongated JT structure, whereas they get the lowest charge for the compressed JT structure. These observations are intuitive in the sense that the filled d-orbitals repel the head-on approaching *n*-donor ligands more when their N-partial charge is higher (i.e., more negative). Then, to gain stability, it is relatively easier to repel two ligands along the *z*-axis (d_*z*^2^_) than four ligands in the xy-plane () for the same d-orbital occupancy. This may qualitatively explain why elongated JT distortion is energetically favorable.

**Table 3:** NPA^73^ Partial Charges for Selected Atoms (CuN_6_) of the Optimized Complex Geometries

		B3LYP/Def2TZVP		CAM-B3LYP/Def2TZVP	
	atom	elongation	compression	elongation	compression
ligand	Cu	+0.937	+0.939	+0.908	+0.940
bpy (1st)	N (axial)	–0.454	–0.436	–0.451	–0.443
	N	–0.449	–0.444	–0.444	–0.450
bpy (2nd)	N	–0.449	–0.444	–0.444	–0.450
	N (axial)	–0.454	–0.436	–0.451	–0.443
bpy (3rd)	N	–0.434	–0.455	–0.429	–0.461
	N	–0.434	–0.455	–0.429	–0.461

### MO Analysis

**Figure 7:**
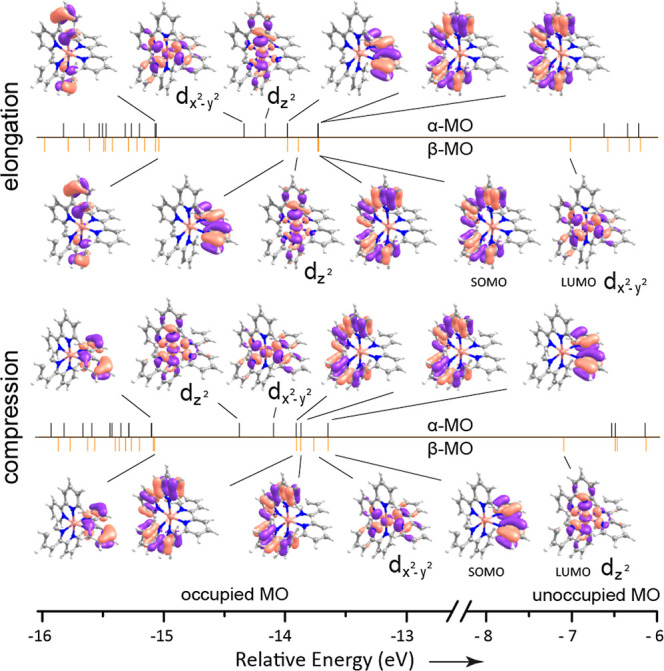
Molecular orbital (α, β-electrons) diagram including key MOs (isosurface value = 0.0432) of the optimized geometries showing elongation (top) and compression (bottom) conformers. Molecules shown with orientation as in Figure 2. The labels d_*z*^2^_,  are qualitative and solely based on visual inspection.

Figure 7 presents the frontier molecular orbitals of both conformers with their energetic ordering, which clearly reshuffles between the two conformers. The singly occupied MO (SOMO) is localized on two bpy ligands for the JT elongated conformer, while on a single bpy for the compressed conformer. Due to their dominant localization on the ligands, they have a smaller contribution to the formation of Cu–N bonds than the MOs denoted d_*z*^2^_ and . The SOMO–LUMO gap for the α-orbitals is 7.11 eV for both conformers; for the β orbitals, it is 6.71 and 6.55 eV for elongation and compression, respectively, suggesting that electronic excitation energies would vary slightly between conformers, which may contribute to the broadness of UV-spectra.^12^ The occupancy of the e_g_ orbitals in the elongated conformer is . A large electron density therefore occurs along the *z*-axis because of the doubly occupied d_*z*^2^_ orbital, reflected in the increased repulsion between the metal and the two axial N atoms. On the other hand, the occupancy of the same e_g_ set in the compressed conformer is , so that a larger electron density occurs in the *xy*-plane. Hence, the energetic ordering of the d-orbitals is important in shaping the conformers. Averaging the orbital energies from the unrestricted calculation in Figure 7 over α and β spin–orbitals, indeed reproduces the swapped ordering of  and d_*z*^2^_ orbitals in the elongated and compressed structures that is qualitatively indicated in Figure 1.^2−4^

## Conclusion

We recorded the IRMPD spectrum of the *m*/*z-*isolated [Cu(2,2′-bipyridine)_3_]^2+^ ion in the gas phase employing the free-electron laser FELIX coupled to a modified quadrupole ion trap mass spectrometer. The IRMPD spectrum is of good quality, showing sharp, well-resolved vibrational bands of 10–15 cm^–1^ bandwidths (fwhm). Computed spectra for the two nearly iso-energetic JT-distorted conformers are nearly identical, but show sensitive differences, especially in the medium-intensity feature near 1015 cm^–1^ that constitutes a convolution over several closely spaced Cu–N stretch normal modes. As the relative bond lengths of the two axial Cu–N bonds versus the four equatorial Cu–N bonds reverse in the two JT-distorted conformers, the convoluted band profile of the 1015 cm^–1^ feature is that of a partly resolved double band with asymmetric intensity distribution, with the lower-intensity component on the low-frequency side for the elongated conformer and on the high-frequency side for the compressed conformer. The frequency splitting between the high and low intensity components is about 10–15 cm^–1^, of the same order of the spectral resolution in our experiment.

The experimental spectrum near 1015 cm^–1^ displays a slightly broadened feature, without signs of a splitting into two components. Hence, one is tempted to suggest that an admixture of the two computed spectral profiles could best reproduce the experimentally observed profile. Furthermore, the computed isomerization barrier suggests that kinetic trapping of the higher-energy conformer may prevent thermalization. In addition, molecular orbitals and their occupancies as well as computed partial charges derived from our calculations confirm the well-known qualitative picture of JT distortion in octahedral metal-tris–bipyridine complexes with uneven occupation of the e_g_ (, d_*z*^2^_) orbitals.

However, computed spectral differences between the JT elongated and JT compressed species are small and even though the experimental spectral resolution is good (for an IRMPD spectrum), a definite conclusion on which of the two conformers (or both) are present in the ion population cannot be established. We conclude that the coexistence of the higher-energy compressed conformer cannot be excluded and that further investigation at improved spectral resolution, possibly via cryogenic ion spectroscopy, is warranted.

## Supporting Information Available

IR spectrum of neutral bpy; frontier orbitals at the TS geometry; partial charge information (PDF)
