# Supplementary material for: Infrared Ion Spectroscopy of Gaseous [Cu(2,2′-Bipyridine)3]2+: Investigation of Jahn–Teller Elongation Versus Compression
Source: J Phys Chem A. 2025 Jan 28;129(5):1318–27. doi: 10.1021/acs.jpca.4c07019 (PMC11808781; doi:10.1021/acs.jpca.4c07019)
Supplement: Supplementary file 1 — jp4c07019_si_001.pdf [file jp4c07019_si_001.pdf]

Supplementary information

Infrared Ion Spectroscopy of Gaseous $[Cu(2,2'\text{-Bipyridine})_3]^{2+}$: Investigation of Jahn-Teller Elongation versus Compression

Musleh Uddin Munshi[a,*], Giel Berden[b], Jos Oomens[b, c,*]

[a]Department of Chemistry, Sogang University, Seoul 04107, Republic of Korea,

e-mail: musleh@sogang.ac.kr

[b]Radboud University, Institute for Molecules and Materials, FELIX Laboratory, Toernooiveld 7,

6525 ED Nijmegen, The Netherlands

[c]University of Amsterdam, Science Park 904, 1098XH Amsterdam, The Netherlands,

e-mail: jos.oomens@ru.nl

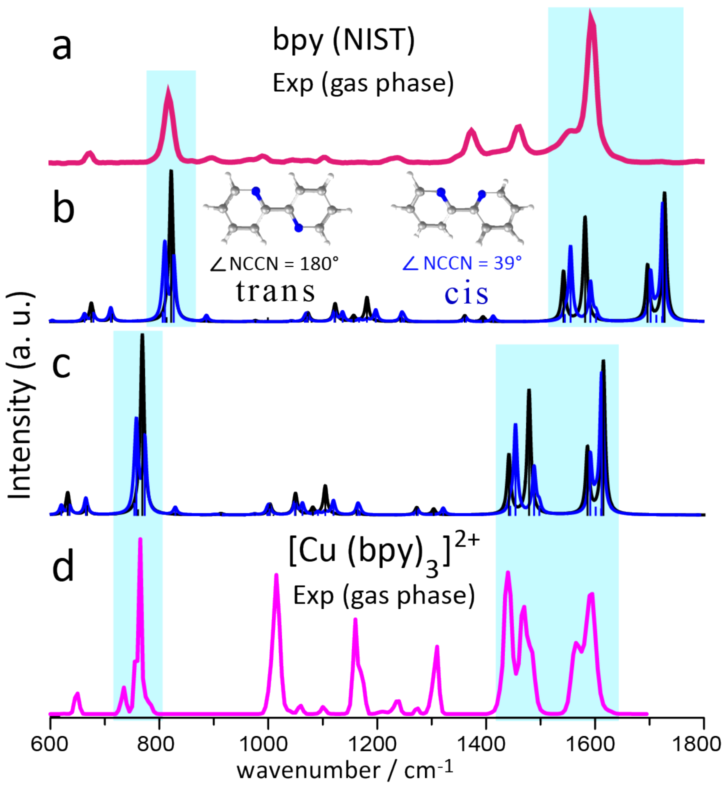

Figure S1 a) Experimental IR spectrum of gaseous 2,2'-bipyridine (bpy) collected from National Institute of Standard and Technology (NIST) (webbook.nist.gov). B3LYP/def2TZVP calculated harmonic IR spectra of trans and cis forms of bpy are shown with a uniform frequency scaling of 1.06 (b) and 0.98 (c). Optimized geometries are provided in panel (b). The IRMPD spectrum of the $[Cu(bpy)_3]^{2+}$ ion recorded in this work is shown in panel (d). Significant IR band shifts (shaded) are observed between neutral free bpy and metal-bound bpy, perhaps related to the sigma donation of electrons to the metal ion, weakening the resonance stabilization of the bpy ligands.

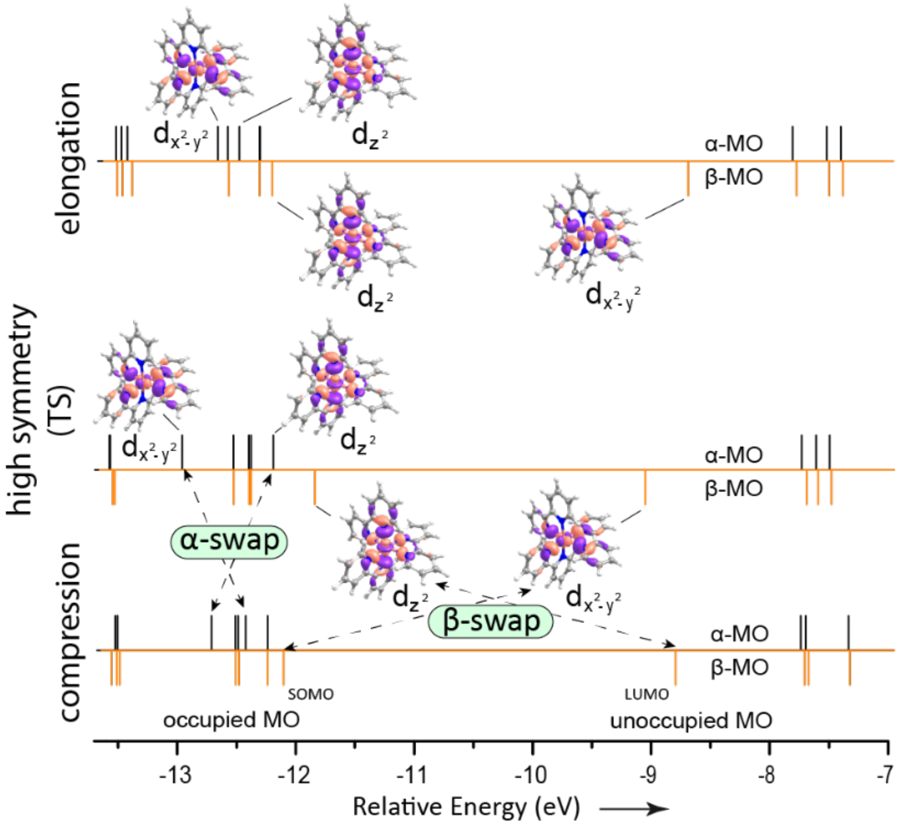

Figure S2 Molecular orbital (α and β-electrons) diagram of B3LYP/def2TZVP optimized geometries of $[Cu(bpy)_3]^{2+}$ in the elongated conformer, the near-D_3 symmetry TS and the compressed conformer. Key MOs are shown with an isosurface value of 0.0432. Molecules shown with same orientation as in Figure 2.

Table S1 Calculated partial charges from a natural population analysis (NPA) for the transition state geometry at the B3LYP/def2TZVP level.

		D_3 symmetry (TS)	Restricted geometry (TS)
	bond	NPA	NPA
bpy (1st)	Cu—N(axial)	-0.464	-0.450
	Cu—N	-0.438	-0.425
bpy (2nd)	Cu—N	-0.438	-0.450
	Cu—N(axial)	-0.464	-0.450
bpy (3rd)	Cu—N	-0.432	-0.425
	Cu—N	-0.432	-0.456
